# The Responsibility of Sexual Pervert

**Published:** 1892-04

**Authors:** 


### THE RESPONSIBILITY OF SEXUAL PERVERT.

In a discussion of Sexual Perversions as related to insanity (Med. Bulletin, March, '92), Dr. E. C. Mann, after calling attention to the case of a highly educated, refined, virtuous and apparently normal minded lady patient, who suffers from epileptic vertigo, generally manifesting itself in connection with the menstrual epoch, asserts that she has had strong impulsions of disease towards crime in the form of suicide; but when these impulsions pass off, she is cheerful, sunshiny, and is a devoted wife and daughter. "Yet," suggests the writer, "it must be a very simple thing for a homicidal to take the place of the suicidal impulse, and, if she should ever in the future give way to such an impulse, the public and her friends would be greatly astonished, and perhaps, refuse to believe her responsible for it, as they have never seen any indication of mental mischief. Every physician, however, can see that the question here should be, not could she distinguish between right and wrong at the time of the commission of the act, but could she help it? Could she avoid the compulsion of disease toward crime? Was the act the outcome and product of the epilepsy? No act which is the product of disease of the body affecting the mind by deranging its functions and causing a suspension or impairment of the healthy intellect, the emotions or the will, can be construed into a crime; and physicians should always voice this in the court room when they have to give their professional opinion. It is time that the laws should be amended to keep pace with medical science, and the absurd right-and-wrong-test of insanity, which is no test at all, should be relegated to the dark ages where it properly belongs."

This is all no doubt scientifically accurate, but we doubt not that a wide-spread knowledge of it by the medical profession, the judiciary, and the public would result in great increase in the sum total of criminality under the cloak of such protective doctrine, for, not even including in our estimate those who would take advantage of the protection without the justification of possessing any such pathological condition, the real subjects of it are no doubt deterred to a certain extent, many times, from committing crimes, though the impulsion be present, because of their accountability under existing laws.—(*Medical Fortnightly,*) March 15, 1892–p. 172.

